# Reduced PTEN expression in the pancreas overexpressing transforming growth factor-beta 1

**DOI:** 10.1038/sj.bjc.6600031

**Published:** 2002-01-21

**Authors:** M P A Ebert, G Fei, L Schandl, C Mawrin, K Dietzmann, P Herrera, H Friess, T M Gress, P Malfertheiner

**Affiliations:** Department of Gastroenterology, Hepatology and Infectious Diseases, Otto-von-Guericke University, Leipzigerstr. 44, D-39120 Magdeburg, Germany; Institute of Neuropathology, Otto-von-Guericke University, D-39120 Magdeburg, Germany; Department of Morphology, University of Geneva, CH-1211 Geneva, Switzerland; Department of Visceral and Transplantation Surgery, University of Berne, CH-3010 Berne, Switzerland; Department of Internal Medicine I, University of Ulm, D-89069 Ulm, Germany

**Keywords:** pancreas, TGF beta, transformation, tumour, MMAC1

## Abstract

*PTEN* is a candidate tumour suppressor gene and frequently mutated in multiple cancers, however, not in pancreatic cancer. Recently, it has been demonstrated that PTEN expression is regulated by TGF-β1. Using TGF-β1 transgenic mice (*n*=7) and wildtype littermates (*n*=6), as well as pancreatic tissues obtained from organ donors (*n*=10) and patients with pancreatic cancer (*n*=10), we assessed the expression of PTEN by means of immunohistochemistry and semiquantitative PCR analysis. In addition, PANC-1 cells were treated with TGF-β1 *in vitro* and the levels of PTEN mRNA were determined in these cells. In human pancreatic cancers PTEN mRNA levels were significantly decreased (*P*<0.05). In addition, in the pancreas of TGF-β1 transgenic mice the expression of PTEN was significantly reduced (*P*<0.01), as compared to wildtype littermates and incubation of PANC-1 cells with TGF-β1 decreased PTEN mRNA levels after 24 h. Inasmuch as TGF-β1 decreases PTEN expression in human pancreatic cancer cells and human pancreatic cancers overexpress TGF-β1, the reduced expression of PTEN in pancreatic cancer may be mediated by TGF-β1 overexpression. Thus, although *PTEN* is not mutated in pancreatic cancers, the reduction of its expression may give pancreatic cancer cells an additional growth advantage.

*British Journal of Cancer* (2002) **86**, 257–262. DOI: 10.1038/sj/bjc/6600031 www.bjcancer.com

© 2002 The Cancer Research Campaign

## 

Pancreatic cancer is an aggressive cancer with an increasing incidence and poor survival (Warshaw and Fernandez-Del Castillo, 1992; Hahn and Schmiegel, 1998). Less than 5% of patients with pancreatic adenocarcinoma survive more than 5 years. The reason for this poor survival is, among others, the presence of advanced stages, the poor therapeutic options and the current incomplete knowledge concerning the pathogenesis and biology of the tumour (Warshaw and Fernandez-Del Castillo, 1992; Hahn and Schmiegel, 1998). Recently, the molecular biology and the pathogenesis of this cancer has been studied extensively, leading to the identification of *K-ras* mutations or the frequent inactivation of tumour suppressor genes, such as *p53*, *p16* or *Smad4* (Warshaw and Fernandez-Del Castillo, 1992; Hahn and Schmiegel, 1998). However, the exact molecular mechanisms of its pathogenesis remain largely unknown. We and others have reported the frequent overexpression of oncogenes and growth factors, such as EGF, FGF, VEGF and PDGF in pancreatic cancers along with the overexpression of their respective receptors (Korc *et al*, 1992; Ebert *et al*, 1995; Kornmann *et al*, 1997; Fujimoto *et al*, 1998). Furthermore, growth factors of the TGF-β superfamily are also overexpressed in this tumour and TGF-β1 overexpression is associated with poor survival in these patients (Friess *et al*, 1993). Although, TGF-β1 exerts an inhibitory effect on cancer cell growth *in vitro,* it also stimulates the synthesis of the extracellular matrix and has been implicated in the regulation of cell differentiation, angiogenesis, immunosuppression and fibrosis (Massagué, 1996; Korc, 1998; Löhr *et al*, 2001).

Recently, *PTEN*, a tumour suppressor gene which is located on chromosome 10q23, was identified by positional cloning (Steck *et al*, 1997; Li *et al*, 1997b). Several groups have reported loss of heterozygosity, mutation or deletion of the gene in different cancers, including glioblastoma, prostate, lung and breast carcinoma and altered expression of PTEN was also detected in various precancerous lesions (Steck *et al*, 1997; Li *et al*, 1997b; McMenamin *et al*, 1999; Perren *et al*, 1999; Sano *et al*, 1999; Mutter *et al*, 2000). PTEN has homology to a protein tyrosine phosphatase and has the activity of a dual-specificity phosphatase (Myers *et al*, 1997). In addition, recent studies indicate that PTEN may function as a mediator of the phosphatidylinositol (PI3′) kinase pathway (Maehama and Dixon, 1998). These findings, along with the description of germline mutations and deletions of *PTEN* in two hereditary cancer predisposition diseases (Liaw *et al*, 1997; Marsh *et al*, 1997; Nelen *et al*, 1997), i.e. Cowden Disease and the Bannayan-Zonana-syndrome, point to a role of *PTEN* as a tumour suppressor gene in the pathogenesis of malignant tumours. However, *PTEN* mutations or deletions do not seem to be present in pancreatic cancer (Sakurada *et al*, 1997; Okami *et al*, 1998). Since PTEN expression is regulated by TGF-β1 (Li *et al*, 1997a), we assessed PTEN mRNA levels in human pancreatic cancers by RT–PCR analysis and immunohistochemistry. In addition, we determined the mRNA levels of PTEN in a model of TGF-β1 overexpressing transgenic mice which develop pancreatic fibrosis and studied the mRNA levels of PTEN in a pancreatic cancer cell line following incubation with TGF-β1.

## MATERIALS AND METHODS

The following products were purchased: Taq polymerase from Gibco-BRL (Eggenstein, Germany). Oligonucleotides were purchased from MWG-Biotech. (Ebersberg, Germany). All other chemicals and reagents were of molecular biology grade and were purchased from Sigma Chemical (Deisenhofen, Germany).

### Transgenic mice

Transforming growth factor beta-1 overexpressing transgenic mice were generated by the introduction of the rat insulin II gene promotor fused to the murine TGF-β1 cDNA. The fusion fragment was inserted in the *Eco*RI site of pBS containing the human growth hormone (Sanvito *et al*, 1995). Seven transgenic animals were used for this study; control animals were of the BL6 strain. Transgenesis was determined on tail DNA by dot blot or Southern hybridization. All animals were maintained on laboratory chow and tap water. Animals were housed and treated in accordance with appropriate guidelines. After 3–9 months animals were sacrificed, the pancreas was removed and tissues were snap frozen in liquid nitrogen. In all transgenic mice the pancreas exhibited massive fibrosis, as previously described (Sanvito *et al*, 1995).

### Tissue samples

Pancreatic cancer tissues (six female, four male) were obtained from patients undergoing pancreatic surgery. Normal pancreatic tissues were obtained from 10 individuals (five female) through an organ donor programme. The median age of the patients with pancreatic cancer was 61.5 years (range, 48–73 years). The median age of the organ donors was 42 years (range, 37–47 years). Immediately following surgical removal, all tissue samples were either fixed in Bouin's solution or frozen in liquid nitrogen. All cancer tissue samples were graded independently by a pathologist, and classified histologically as adenocarcinoma of the exocrine pancreas. This study was approved by the Ethics Committee of the University of Berne, Switzerland.

### Cell line

PANC-1 cells were cultured in DMEM medium (Gibco-BRL, Gaithersburg, MA, USA) supplemented with 10% foetal calf serum (FCS, Gibco-BRL), penicillin (100 U ml^−1^), and streptomycin (100 U ml^−1^) at 37°C in 5% CO_2_ atmosphere. To analyze the effect of TGF-β1 on cell morphology pancreatic cancer cell lines were grown to 70% confluence in DMEM containing 10% FCS. Afterwards, cells were washed twice in serum free medium, starved for 24 h in serum free medium and finally treated for 12 and 24 h with 10 ng ml^−1^ TGF-β1 (R&D, Wiesbaden, Germany) or medium alone (Geng *et al*, 1999). Thereafter total RNA was extracted using the RNAclean kit (AGS, Heidelberg, Germany) as indicated by the manufacturer.

### RNA extraction

Total RNA was extracted from the human pancreatic tissues and the pancreas of the transgenic mice by the acid guanidinium-thiocyanate method, as previously described (Ebert *et al*, 1994).

### Polymerase chain reaction

Oligonucleotide primers were purchased from MWG-Biotech. (Ebersberg, Germany). cDNAs were synthesized from total RNA (1 μg sample^−1^) isolated from human and mouse pancreatic tissues and the cancer cell line, using oligodeoxythymidylate and reverse transcriptase. Following inactivation, 1 μl of the reaction mixture were incubated in buffer containing 0.2 mM concentrations of dATP, dCTP, dGTP, dTTP, 0.2 μM concentrations each of oligonucleotide primers, 3 mM MgCl_2_ and a 10× buffer consisting of 200 mM Tris-HCl (pH 8.0), 500 mM KCl, and 1 U Taq polymerase. PCR primers were designed to amplify a region spanning from 117 to 741 of the *PTEN* gene (F1, 5′-CAGAAAGACTTGAAGGCGTAT-3′ and B1, 5′-AACGGCTGAGGGAACTC-3′), as previously described (Sano *et al*, 1999). The 624 bp fragment contains a *Nsi*I restriction site allowing to distinguish it from the *PTEN* pseudogene (Sano *et al*, 1999). The primers designed to amplify enolase comprise a region from 77 to 532 bp of the coding region of the *enolase* gene (E1, 5′-TGGCAGGACTTCAGA-3′; E2, 5′-AGTGGCTAGAAGTTCACC-3′). For semiquantitative analysis of PTEN mRNA levels in the pancreas of transgenic and wildtype mice, the primer F1 was used in conjunction with a different antisense primer (H1, 5′-TCTAGGGCCTCTTGTGCCTTT-3′) for the amplification of a 622 bp fragment of mouse PTEN (Sano *et al*, 1999). Again, digestion with *Nsi*I allowed differentiation from the *PTEN* pseudogene (Sano *et al*, 1999). In addition, primers specific for rat glyceraldehyde-3-phosphate dehydrogenase (GAP) mRNA (G1,G2) were also added to the reaction in order to assess the PTEN mRNA levels in the murine pancreas semiquantitatively. Primers were chosen as previously reported (Ebert *et al*, 1999): G1, 5′-GCTGGATCCTTCATTGACCTCAACTAG-3′; G2, 5′-CGAGAATTCATACCAGGAAATGAGC-3′. PCR amplification was performed after an initial denaturation of 3 min at 94°C, followed by 40 cycles of 45 s at 94°C, 45 s at 54°C and 1 min at 72°C, and finally 10 min of final elongation at 72°C. The PCR products were treated with *Nsi*I at 37°C for 2 h and then separated on 1.5% agarose gel (Ebert *et al*, 1994). The level of PTEN mRNA was analyzed densitometrically from the agarose gel and was standardized to the respective enolase or GAP mRNA level. The PTEN:enolase or PTEN:GAP ratio was calculated and was analyzed by the *t*-test (Siegel, 1956).

### Immunohistochemistry

**Figure 1:**
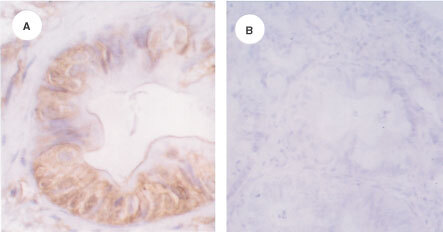
Immunohistochemical analysis of PTEN expression in pancreatic cancer. (**A**) In human pancreatic cancers PTEN immunoreactivity was present in some of the cancer cells. (**B**) Sections incubated with the anti-PTEN antibody and the blocking peptide exhibited no PTEN immunoreactivity.

The presence of human PTEN was assessed using paraffin-embedded tissue sections obtained from 10 patients with pancreatic cancer undergoing pancreatic surgery. The human tissues were fixed in Bouin's solution and paraffin embedded. The anti-PTEN antibody (n-19) is an affinity-purified goat polyclonal antibody raised against a peptide mapping at the amino-terminus of human PTEN and was used at a dilution of 1:800 (Santa Cruz, CA, USA) (Steck *et al*, 1997; Sano *et al*, 1999). In addition, sections were also incubated with the anti-PTEN antibody C-20, which is a goat polyclonal antibody raised against a peptide mapping at the carboxy-terminus of human PTEN (Santa Cruz, CA, USA). Paraffin sections (4 μm thick) were deparaffinized and rehydrated. For negative controls, the primary antibodies were omitted and/or preimmune serum was used. Furthermore, the anti-PTEN antibody n-19 was also incubated with its respective blocking peptide which resulted in no specific immunoreactivity in the immunohistochemical analysis, demonstrating the specificity of the anti-PTEN antibody (Figure 1

). Endogenous peroxidase activity was inhibited by immersing the sections in 0.3% H_2_O_2_ for 30 min. The sections were incubated with the antiserum at 37°C for 1 h and washed with PBS buffer. The reaction was detected using the standard streptavidin-peroxidase technique (LSAB kit, DAKO Hamburg, Germany). The analysis was performed according to the manufacturer's recommendations and all reactions were performed at 23°C. Finally, the sections were counterstained with Mayer's hematoxylin (Ebert *et al*, 1994).

### Statistical analysis

The *t*-test was used to determine statistical difference. A *P* value of less than 0.05 was considered statistically significant (Siegel, 1956).

## RESULTS

**Figure 2:**
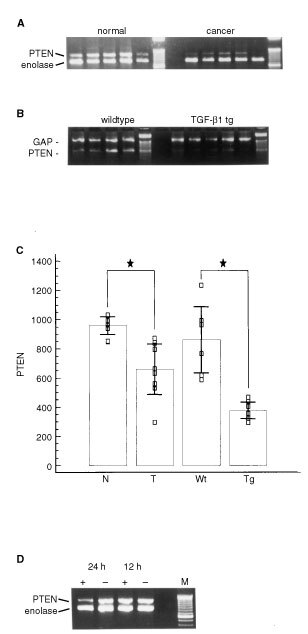
RT–PCR analysis of PTEN mRNA levels in a pancreatic cancer cell line, in human and murine pancreas. (**A**) Semiquantitative analysis revealed decreased expression of PTEN mRNA in pancreatic cancers (right) as compared to the normal pancreas (left). Last lane, DNA ladder. (**B**) In transgenic mice overexpressing TGF-β1 (TGF-β1 tg) PTEN mRNA levels were also decreased as compared to wildtype littermates (wildtype). Last lane, DNA ladder. (**C**) Densitometric analysis confirmed a significant reduction of PTEN mRNA levels in pancreatic tumours (T) as compared to the normal pancreas (N) and in TGF-β1 transgenic mice (Tg) as compared to wildtype mice (Wt). Mean ±s.d.; □, PTEN mRNA levels as determined by densitometric analysis and standardization against their respective enolase mRNA or GAP mRNA levels; ★, *P*<0.05. (**D**) Incubation of PANC-1 cells with TGF-β1 led to a significant reduction of PTEN mRNA levels after 24 h, however not after 12 h. +, addition of TGF-β1; −, control without agonist addition; M, DNA ladder.

In human pancreatic cancers RT–PCR analysis revealed the presence of PTEN mRNA in all samples (Figure 2A

). PTEN immunoreactivity was also detected in some of the cancer cells (Figure 1A), whereas some cancer cells were devoid of PTEN immunoreactivity. Immunohistochemical analysis of pancreatic cancer tissue sections after incubation of the primary antibody with the blocking peptide exhibited no specific immunoreactivity (Figure 1B).

In order to determine the levels of PTEN mRNA in the pancreas of individuals without pancreatic disease and in the pancreas of patients with pancreatic adenocarcinoma we coamplified cDNA fragments encoding PTEN mRNA and enolase mRNA. In comparison with the PTEN mRNA levels in the normal pancreas obtained from organ donors without malignant disease of the pancreas, we found a significant reduction of PTEN mRNA levels in the cancer tissues (*P*<0.01) (Figure 2A,C).

Since PTEN expression is regulated in part by TGF-β1 which is overexpressed in pancreatic cancer cells, we studied the levels of PTEN mRNA in the pancreas of transgenic mice overexpressing TGF-β1. These mice exhibit massive fibrosis of the exocrine pancreas through overexpression of TGF-β1 under the control of a rat insulin II gene promotor. Using semiquantitative RT–PCR, we determined the PTEN mRNA levels in the pancreas of seven transgenic and six wildtype mice (Figure 2B). All mice expressed PTEN mRNA and GAP mRNA in the pancreas, as determined by RT–PCR analysis of cDNAs generated from total RNA. After quantification of PTEN mRNA levels using semiquantitative RT–PCR we detected a significant reduction of PTEN mRNA levels in the pancreas of the TGF-β1 overexpressing mice as compared to the normal pancreas (*P*<0.05) (Figure 2B,C).

Finally, in order to demonstrate a direct effect of TGF-β1 on PTEN mRNA levels in pancreatic cancer cells *in vitro*, we incubated PANC-1 cancer cells with TGF-β1 (10 ng ml^−1^) for 12 and 24 h. Semiquantitative RT–PCR analysis was performed via coamplification of PTEN and enolase mRNA. PANC-1 cells expressed abundant PTEN mRNA (Figure 2D). Using this semiquantitative RT–PCR analysis we observed no reduction of PTEN mRNA levels after 12 h of TGF-β1 treatment (+) as compared to the untreated control (−). However, after 24 h incubation with TGF-β1 a more than 60% reduction of PTEN mRNA levels was observed in PANC-1 cancer cells (Figure 2D, left).

## DISCUSSION

The tumour suppressor gene *PTEN*, located on chromosome 10q23, encodes a protein tyrosine phosphatase and is frequently deleted and mutated in various malignancies, including glioblastomas, breast and prostate cancer (Steck *et al*, 1997; Li *et al*, 1997b; Sano *et al*, 1999; Perren *et al*, 2000). Furthermore, previous studies have reported frequent loss of heterozygosity at chromosome 10q23 in endometrial and thyroid cancers (Simpkins *et al*, 1998). This gene is considered to act as a tumour suppressor gene in the pathogenesis of multiple cancers for several reasons: (i) This gene is frequently mutated in tumours arising in patients with Cowden disease and the Bannayan-Zonana syndrome, which are both characterized as hereditary syndromes leading to multiple hamartomas in the case of Cowden disease and both exhibit an increased risk of developing breast, thyroid and skin cancer (Liaw *et al*, 1997; Lynch *et al*, 1997; Marsh *et al*, 1997; Nelen *et al*, 1997). (ii) PTEN has been shown to act against phosphatidylinositol phosphates, pointing to a role of PTEN as a mediator of the phosphatidylinositol kinase pathway (Myers *et al*, 1997; Maehama and Dixon, 1998). (iii) Expression of PTEN is associated with growth inhibition and inhibition of transformation of epithelial cells and PTEN exerts an inhibitory effect on the regulation of cell spreading and migration. (iv) Furthermore, it has been shown to induce apoptosis *in vitro* and *in vivo* (Stambolic *et al*, 1998; Davies *et al*, 1999). In summary, the various biological aspects of PTEN function support a role of *PTEN* as a tumour suppressing gene *in vivo* and *in vitro*.

While we and others have identified a high frequency of growth factor overexpression in pancreatic cancer, the molecular mechanisms of growth factor mediated pancreatic tumorigenesis remain largely unknown (Korc *et al*, 1992; Ebert *et al*, 1995; Fujimoto *et al*, 1998; Löhr *et al*, 2001). TGF-β1, the most prominent and well characterized member of the large TGF-β superfamily, is highly expressed in pancreatic cancers (Friess *et al*, 1993). Although it exerts a growth inhibitory effect on pancreatic cancer cells *in vitro*, its overexpression is associated with poor survival in patients with advanced pancreatic cancer. In addition, the two other mammalian isoforms of TGF-β, i.e. TGF-β2 and TGF-β3, are also overexpressed in pancreatic cancer (Friess *et al*, 1993; Satoh *et al*, 1998) and human pancreatic cancers also express high levels of TGF-β receptor II (TβR-II). In contrast, TβR-I is not aberrantly expressed in pancreatic cancers (Korc, 1998).

The molecular mechanism of the resistance of pancreatic cancers to the growth inhibitory action of TGF-βs, however, is not fully understood. TGF-β1 overexpression may result from the decreased expression of its receptors (Korc, 1998). Furthermore, *T*β*R-II* might be inactivated by mutation which has been reported in colon and gastric cancers of the replication error positive phenotype. For instance, in the MiaPaCa-2 pancreatic cancer cell line low levels of *T*β*R-II* expression and mutation of the kinase domain have been reported (Korc, 1998). In another study, mutations of the polyadenine tract of *T*β*R-II* and mutations in the kinase domain of the *T*β*R-II* gene have been identified (Korc, 1998). In addition, the inactivation of down-stream targets of TGF-β induced signalling pathways, such as *Smad4* which is mutated or deleted in a high percentage of pancreatic cancers, may lead to a subsequent induction of TGF-β1 expression in pancreatic cancers (Hahn and Schmiegel, 1998). Nonetheless, the overexpression of TGF-β1 in pancreatic cancer has an important role in the pathogenesis of this malignancy (Friess *et al*, 1993; Löhr *et al*, 2001). Through the enhancement of tumour angiogenesis, the induction of the epithelial-mesenchymal interaction, and the suppression of an adequate immune response TGF-β1 may contribute to pancreatic tumour development (Friess *et al*, 1993; Massagué, 1996; Löhr *et al*, 2001). Recently, several molecular alterations which are induced by TGF-β1 in pancreatic cancer cells *in vitro* have been identified. Thus, the induction of PDGFs and cyclin D1 in pancreatic cancer cells by TGF-β1 has been reported (Ebert *et al*, 1995; Kornmann *et al*, 1999). Our current study may further help to understand how this growth factor may contribute to pancreatic cancer pathogenesis, despite its growth inhibitory effect on pancreatic cancer cells *in vitro*. In this study we found significantly decreased levels of PTEN mRNA in pancreatic cancers. Since two reports have demonstrated that *PTEN* mutations or deletions are not present in pancreatic cancers (Sakurada *et al*, 1997; Okami *et al*, 1998), other molecular mechanisms must contribute to its altered expression in pancreatic cancer. In a study by Li *et al* (1997a) the protein tyrosine phosphatase PTEN was found to be rapidly down-regulated by TGF-β1 in the HaCaT keratinocyte cell line. The incubation of TGF-β1 at a concentration of 2 ng ml^−1^ to actively growing HaCaT cells was associated with a marked reduction of PTEN mRNA levels occurring within 2 h after addition of the cytokine. While PTEN expression therefore seems to be regulated in part by TGF-β1 (Li *et al*, 1997a), we raised the hypothesis that the overexpression of TGF-β1 in pancreatic cancers may reduce the expression of PTEN which in turn may give these cells an additional growth advantage. First, we determined the levels of PTEN mRNA in human pancreatic cancers and found a significant reduction of PTEN expression in pancreatic cancer cells. In order to demonstrate that this reduction is mediated by TGF-β1, we analyzed the expression of PTEN in a transgenic model of TGF-β1 overexpressing mice. The pancreas of these transgenic mice exhibits massive fibrosis (Sanvito *et al*, 1995). Our analysis revealed a reduction of PTEN mRNA levels in the pancreas of these mice as compared to wildtype littermates. To further substantiate our findings, we incubated PANC-1 pancreatic cancer cells with TGF-β1 and found a dramatic reduction of PTEN mRNA levels in these cells. Interestingly, the reduction of PTEN mRNA levels in the PANC-1 cell line was present after 24 h incubation with TGF-β1 in contrast to the report by Li *et al* (1997a) who found a rapid decline of PTEN mRNA levels within 2 h after addition of TGF-β1. While we only analyzed the PTEN mRNA levels 12 and 24 h after incubation of PANC-1 cells with TGF-β1, we cannot exclude the possibility that a rapid reduction of PTEN mRNA levels had taken place earlier in the pancreatic cancer cells as well, which may have been followed by a restitution of PTEN mRNA levels and a second decrease 24 h after treatment of the cells. Furthermore, the concentrations of TGF-β1 in our study and that by Li *et al* (1997a) were different, so that a dose-dependent effect may also play a role in these findings. Nonetheless our studies confirm that PTEN mRNA levels are regulated and controlled at least in part by TGF-β1, which supports our hypothesis that the overexpression of TGF-β1 in pancreatic cancer may contribute to reduced PTEN mRNA levels in this malignancy. Thus, in conclusion our data strongly support the hypothesis that the decreased expression of this tumour suppressor in pancreatic cancers may result from the overexpression of TGF-β1.

Besides the regulation by TGF-β1, other molecular mechanisms may also contribute to the downregulation of PTEN expression in malignant tumours. Thus, the reduced expression of PTEN may result from hypermethylation of the PTEN promotor, the enhanced degradation of the transcript or the transcriptional inactivation of the gene (Lynch *et al*, 1997; Whang *et al*, 1998). For instance, in Cowden disease, nonsense-mediated degradation of the gene was reported (Liaw *et al*, 1997; Lynch *et al*, 1997; Nelen *et al*, 1997). In addition, in a study using prostate cancer tissues and cell lines, the treatment of the cells with the demethylating agent 5-azadeoxycytidine led to the restoration of PTEN mRNA levels, which reflects a possible role of hypermethylation of the promotor in the regulation of PTEN transcription (Whang *et al*, 1998). We did not study the methylation status of the PTEN promotor in our cancer samples. In fact, our study indicates that TGF-β1 contributes to reduced PTEN mRNA levels in pancreatic cancers.

Thus, despite the fact that PTEN inactivation through *PTEN* mutation or deletion is infrequent in pancreatic cancer, we raise the hypothesis that TGF-β1 overexpression may lead to reduced PTEN mRNA levels in pancreatic cancers which may give these cells an additional growth advantage and, thus, contributes to the aggressive phenotype of this cancer.
